# Advanced Xenograft Model with Cotransplantation of Patient-Derived Organoids and Endothelial Colony-Forming Cells for Precision Medicine

**DOI:** 10.1155/2021/9994535

**Published:** 2021-07-13

**Authors:** Junhye Kwon, Sungryong Oh, Misun Park, Joon Seog Kong, Sunyi Lee, Hyunsook Lee, Younjoo Kim, Kyu-Tae Kang, Ui Sup Shin, Joohee Jung

**Affiliations:** ^1^Department of Radiological & Clinical Research, Korea Cancer Center Hospital, Korea Institute of Radiological and Medical Sciences (KIRAMS), Seoul 01812, Republic of Korea; ^2^College of Pharmacy, Duksung Women's University, Seoul 01369, Republic of Korea; ^3^Duksung Innovative Drug Center, Duksung Women's University, Seoul 01369, Republic of Korea; ^4^Department of Pathology, Korea Cancer Center Hospital, KIRAMS, Seoul 01812, Republic of Korea; ^5^Department of Internal Medicine, Korea Cancer Center Hospital, KIRAMS, Seoul 01812, Republic of Korea; ^6^Department of Surgery, Korea Cancer Center Hospital, KIRAMS, Seoul 01812, Republic of Korea

## Abstract

Preclinical evaluation models have been developed for precision medicine, with patient-derived xenograft models (PDXs) and patient-derived organoids (PDOs) attracting increasing attention. However, each of these models has application limitations. In this study, an advanced xenograft model was established and used for drug screening. PDO and endothelial colony-forming cells (ECFCs) were cotransplanted in NRGA mice (PDOXwE) to prepare the model, which could also be subcultured in Balb/c nude mice. Our DNA sequencing analysis and immunohistochemistry results indicated that PDOXwE maintained patient genetic information and tumor heterogeneity. Moreover, the model enhanced tumor growth more than the PDO-bearing xenograft model (PDOX). The PDO, PDOXwE, and clinical data were also compared in the liver metastasis of a colorectal cancer patient, demonstrating that the chemosensitivity of PDO and PDOXwE coincided with the clinical data. These results suggest that PDOXwE is an improvement of PDOX and is suitable as an evaluation model for precision medicine.

## 1. Introduction

Precision medicine, which encompasses personalized medicine, is important for the implementation of optimized anticancer therapy. Moreover, preclinical evaluation models are indispensable in screening for the sensitivity of anticancer drugs. Patient-derived organoids (PDOs) have been established as an *in vitro* model for various cancers [1]. This model embodies the function and genetic information of a patient's tissue and could be maintained for an expanded period [2]. PDOs have emerged as a high-throughput screening system in anticancer drug prognosis and development [3]. Although PDOs recapitulate the features of tissues, they cannot emulate the tumor microenvironment. Therefore, several anticancer drugs that interrupt the crosstalk between cancer cells and surrounding cells could not be evaluated using this model. Consequently, *in vivo* animal models are required for an accurate prognosis before clinical application. Recently, patient-derived xenograft models (PDXs) directly transplanted with patient tissue and PDO-bearing xenograft models (PDOXs) have been established [4]. By contrast, PDXs and PDOX can preserve cancer heterogeneity and the genetic information of the patient's tissue, as well as mimic the tumor microenvironment. However, the establishment rate of PDXs remain low [5, 6] because patient tissue is composed of cancer cells and stroma and this ratio is not constant. The PDOX could improve on the disadvantages of PDX. Nevertheless, the PDX and PDOX protocols have not yet been optimized because they differ depending on the cancer type. Furthermore, the time required for the establishment of PDX and PDOX remains a limiting factor in their application on anticancer drug screening.

In this study, an advanced xenograft model was developed using colorectal cancer (CRC) patient-derived tissues to overcome the limitations of PDOX. CRC is known to cause liver metastasis, and its progression can lead to death [7]. Thus, a strategy for the inhibition of CRC progression is important in increasing survival rate. In particular, there is an urgent need for anticancer drugs optimized for accuracy in anticancer therapy applications. Hence, we investigated the sensitivity of anticancer drugs in advanced xenograft models of CRC and liver metastatic CRC.

## 2. Materials and Methods

### 2.1. Human Tissue Acquisition and Patient Treatment

The protocol for this section of the study was approved by the Ethics Committee of the Korea Cancer Center Hospital (approval no. KIRAMS-2017-07-001 and KIRAMS-2017-09-009) and was performed in accordance with the approved guidelines and regulations of the institution. All samples were obtained from patients who provided written informed consent for the use of their tissues. Surgically resected liver metastatic intestinal cancer tissue (LMT) and endoscopic biopsy intestinal cancer tissues were obtained from patients diagnosed with CRC and treated at the Korea Cancer Center Hospital. The collected samples were also histologically verified as adenocarcinoma by a pathologist using hematoxylin and eosin (H&E) staining. The isolation of the tumor epithelium was performed as previously described with minor modifications [8, 9].

For chemotherapy, the LMT patient was treated with an irinotecan-based regimen (FOLFIRI). The FOLFIRI regimen consisted of 180 mg/m^2^ irinotecan, 400 mg/m^2^ bolus 5-fluorouracil (5-FU), and 2400 mg/m^2^ infusional 5-FU every two weeks. The patient's response to chemotherapy was evaluated after every three cycles with computed tomography (CT, Ingenuity, Philips Healthcare, Amsterdam, the Netherlands) as scored using Response Evaluation Criteria In Solid Tumors 1.1.

### 2.2. Organoid Culture

The tumor organoids were isolated as previously described [10]. Briefly, cancer tissues were incubated with collagenase type II (Sigma-Aldrich, Louis, MO, USA), dispase type II (Roche Applied Science, Mannheim, Germany), and Y-27632 (BioVision, Mountain View, CA, USA) for 1 h at 37°C. Isolated cells were washed with PBS and centrifuged at 300 ×g for 3 min. The cells were then embedded in Matrigel (growth factor reduced, phenol red free; Corning, NY, USA) and seeded in 4-well plates, followed by the addition of the culture medium. The composition of the CRC organoid culture medium was 1 × B27 (Gibco, Grand Island, NY, USA), 1.25 mM N-acetyl cysteine (United States Pharmacopeia, Rockville, MD, USA), 50 ng/mL human epidermal growth factor (BioVision), 50 ng/mL human Noggin (Peprotech, Rocky Hill, NJ, USA), 10 nM gastrin (Sigma-Aldrich), 500 nM A83-01 (BioVision), and 100 mg/mL primocin (InvivoGen, San Diego, CA, USA). To prevent anoikis, 10 *μ*M Y-27632 was added to the culture medium during the first 2-3 days. When organoids were >200 *µ*m, they were passaged by pipetting using the Gentle Cell Dissociation Reagent (STEMCELL Technologies, Vancouver, Canada) according to the manufacturer's instructions.

### 2.3. Organoid Viability

LMT organoids in good condition were harvested, passaged, and seeded in 96-well cell culture plates. The organoid density was adjusted to 50–60/10 *μ*L Matrigel with 200 *μ*L culture medium. For drug testing, the organoid culture medium was removed and replaced with a 200 *μ*L drug-containing culture medium: 2.5 mg/mL cetuximab (Erbitux, Merck), irinotecan (I1406, Merck), or oxaliplatin (O9512, Sigma). Organoids were photographed seven days after drug treatment (EVOS FL Cell Imaging System, Thermo Fisher Scientific), and cell viability was also evaluated at seven days by the CellTiter 96 Aqueous One Solution cell assay (Promega, G3580) according to the manufacturer's instructions.

### 2.4. Culture of Human Endothelial Colony-Forming Cells

Endothelial colony-forming cells (ECFCs) were isolated from the adherent mononuclear cell fraction of human peripheral blood using CD31-coated magnetic beads (Invitrogen, MA, USA) as previously described [11]. Isolated ECFCs were expanded on 1% gelatin-coated plates (BD Biosciences, NJ, USA) using an endothelial cell growth medium MV 2 (EGM-MV 2 without hydrocortisone; PromoCell, Heidelberg, Germany) supplemented with 10% fetal bovine serum (Atlas Biologicals, CO, USA) and 1% glutamine-penicillin-streptomycin (Gibco, MA, USA). ECFCs between passages seven and ten were used in all of the experiments. The protocol for this section of the study was approved by the institutional review board of Duksung Women's University (IRB Nos. 2017-002-001 and 2018-007-006).

### 2.5. Animal Handling

All animal experiments were carried out following the protocol approved by the Institutional Animal Care and Use Committee of Duksung Women's University (No. 2019-012-001). Five-week-old female and male NOD/ShiLtJ-Rag2em1AMC (NRGA) mice and Balb/c nude mice were purchased from JUNGA Bio (Gyeonggi, Korea). All animals were acclimated to the animal laboratory of Duksung Women's University for one week prior to any procedural work. The room conditions were maintained at 20°C, 50% humidity, and a 12/12 h light/dark cycle. The diet was provided with drinking water ad libitum.

### 2.6. Organoid-Derived Xenograft Models

Cultured organoids were collected and implanted into the subcutaneous pockets of NRGA mice. For the coimplantation of organoids and ECFCs, ECFCs were prepared at 1 × 106 cells/100 *μ*L in 10% Matrigel (YoungIn Frontier, Korea) and injected subcutaneously around the implanted organoid. To subculture the organoid-derived xenograft model, organoid-derived tumors were isolated and sliced into 1-2 mm^3^ sections. One piece of tumor tissue was subcutaneously implanted into the second generation of Balb/c nude mice (G2). Subsequently, the G2 xenograft mouse models were used to investigate the efficacy of anticancer drugs. Tumor size was measured using a caliper (Mitutoyo Corporation, Japan) three times per week. The tumor volume was calculated as follows:

(1) $$\begin{array}{c} \text{Tumor volume}\left({\text{mm}}^{3}\right)=\frac{\left(\text{longest length}\right)\times {\left(\text{shortest length}\right)}^{2}}{2}. \end{array}$$

When the tumor volume reached approximately 100 mm^3^, the mice were randomly divided into groups (*n* = 5/group).

### 2.7. Immunohistochemistry

To characterize organoids and their tissues of origin, immunohistochemistry was performed using the colorectal marker CDX2 (1 : 200; cat. no. 235R-16; Cellmarque), CK7 (1 : 10000; cat. no. ab181598; Abcam), and CK20 (1 : 500; cat. no. 320M-16; Cellmarque) in 5 *µ*m formalin-fixed paraffin-embedded tissues and organoid sections (28114961). All images were acquired using an OLYMPUS IX73 (Olympus, Germany).

### 2.8. Tumor Organoid DNA Sequencing and Analysis

To analyze the mutational status of patient tissues, organoids, and PDOX tissues, DNA extraction and library construction were performed using the Qiagen Gentra Puregene kit (Valencia, CA, USA) and Agilent SureSelect XT library prep kit (Santa Clara, CA, USA). Deep targeted sequencing using Axen Cancer Panel 2 (170 cancer-related genes, Macrogen) and the NextSeq 500 midoutput system platform (Illumina) was conducted on tumor tissues, organoids, and PDOX samples. Libraries consisting of 150 bp paired-end reads were sequenced by high-throughput sequencing using synthesis technology to a depth coverage of approximately 2000x. An oncoplot was used for the visualization of the mutations of the tissue, organoid, and PDOX.

### 2.9. Drug Treatment

Intraperitoneal injections of the test drugs were administered following this treatment schedule: oxaliplatin (5 mg/kg, three times/week), irinotecan (20 mg/kg, five times/week), and/or cetuximab (10 mg/kg, twice a week).

### 2.10. Statistics

Data are presented as the mean ± standard deviation. Statistical significance was set at *p* < 0.05 and was calculated using Student's *t* test and one-way ANOVA followed by Tukey's post hoc test.

## 3. Results

### 3.1. PDOX Maintains Patient-Derived Properties

**Figure 1:**
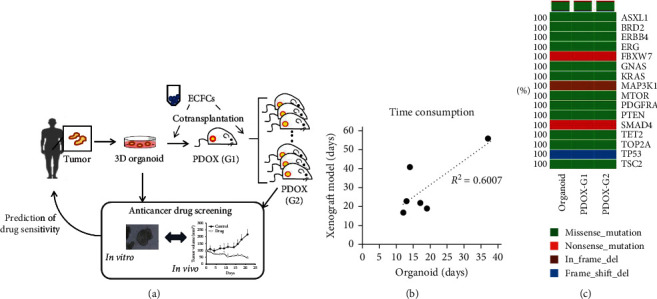
Cancer-patient-derived organoid and organoid-derived xenograft model. (a) Scheme of the *in vitro* and *in vivo* model for precision medicine. The patient-derived organoid (PDO, *in vitro* model) and patient-derived organoid-bearing xenograft models (PDOX, *in vivo* model) are utilized for anticancer drug screening. The advanced xenograft model is a PDOX cotransplanted with endothelial colony-forming cells (ECFCs). For screening, tumor tissues obtained from PDOX (1st generation, G1) were transplanted into Balb/c nude mice (2nd generation, G2). (b) Correlation of the production period between PDO and PDOX. Six cases of colorectal cancer patients were used for production of organoid and xenograft models. (c) Retention of representative gene expression in organoids and PDOX. Sixteen major genes were compared between the organoid and PDOX (G1 and G2).

The sensitivity of anticancer drugs was predicted by screening using the PDO and PDOX models (Figure 1(a)). In our study, we cotransplanted PDO with ECFCs in NRGA mice (G1) and subcultured PDOX (G1) with ECFCs in Balb/c nude mice (G2). First, we investigated whether PDOX maintained the characteristics of PDO. As shown in Figure 1(b), the gene expression of PDO, PDOX (G1), and PDOX (G2) coincided with each other. Moreover, the establishment period of PDO correlated with that of PDOX (*R* = 0.6007) (Figure 1(c)). The establishment period of an *in vivo* model is their limitation in precision medicine applications. Hence, we investigated whether advanced xenograft models can improve the original PDOX.

### 3.2. PDOX with ECFCs Overcomes the Obstacles of PDOX

**Figure 2:**
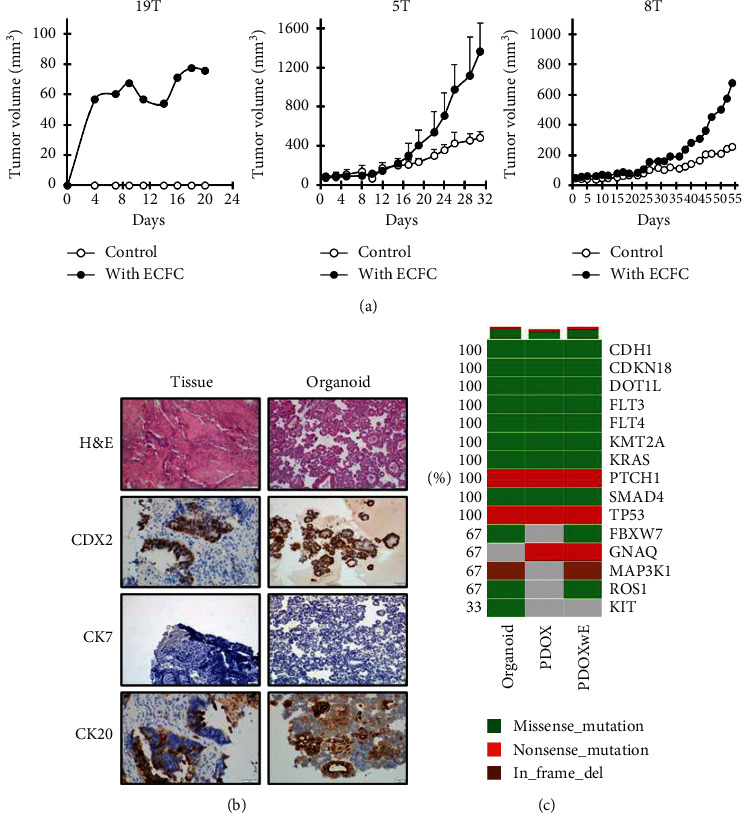
Enhancement of tumor growth in PDOX with ECFCs (PDOXwE). (a) Comparison of tumor growth between PDOX and PDOXwE. PDO only or PDO with ECFCs were transplanted in NRGA mice (G1 xenograft model; 19T patient) and Balb/c mice (G2 xenograft model; 5T patient, *n* = 4/group; 8T patient, *n* = 1/group) ^*∗*^*p* < 0.05 (Student's *t* test). (b) Observation of the 5T patient's tumor tissue and organoid. Sections of the patient's tissue and patient-derived organoid were observed using H&E staining and immunohistochemistry (CDX2, CK7, and CK20). (c) Gene expression of the 5T patient's tumor-derived organoid, PDOX, and PDOXwE. The expression of fifteen major genes was analyzed by DNA sequencing.

The tumor growth of PDOX with ECFCs (PDOXwE) was compared with that of PDOX, because the establishment period of PDOX is an obstacle for its utilization. In 19T-PDO, the establishment of PDOX (G1) failed, but cotransplantation POD with ECFCs showed tumorigenicity (Figure 2(a), left). Furthermore, PDOXwE stimulated tumor growth more than PDOXs in the case of 5T-PDO and 8T-PDO (Figure 2(a), middle and right, respectively). Among them, 5T-PDO was also confirmed to maintain the 5T patient's properties (Figure 2(b)). Additionally, gene expression in PDOXwE coincided with that in PDO (Figure 2(c)). These results indicate that PDOXwE overcomes the obstacle of PDOX by enhancing tumorigenicity and tumor growth while maintaining the advantages of PDOX.

### 3.3. Drug Sensitivity Is Consistent in PDO and PDOXwE

Our results indicate that PDOX drug sensitivity was consistent with that of the patient. The chemotherapeutic efficacy of anticancer drugs was evaluated in PDOXwE and PDO, and the application validity of PDOXwE as an advanced xenograft model is shown in Figure 2. To compare preclinical data with clinical data, we used liver metastatic CRC patient-derived organoids.

**Figure 3:**
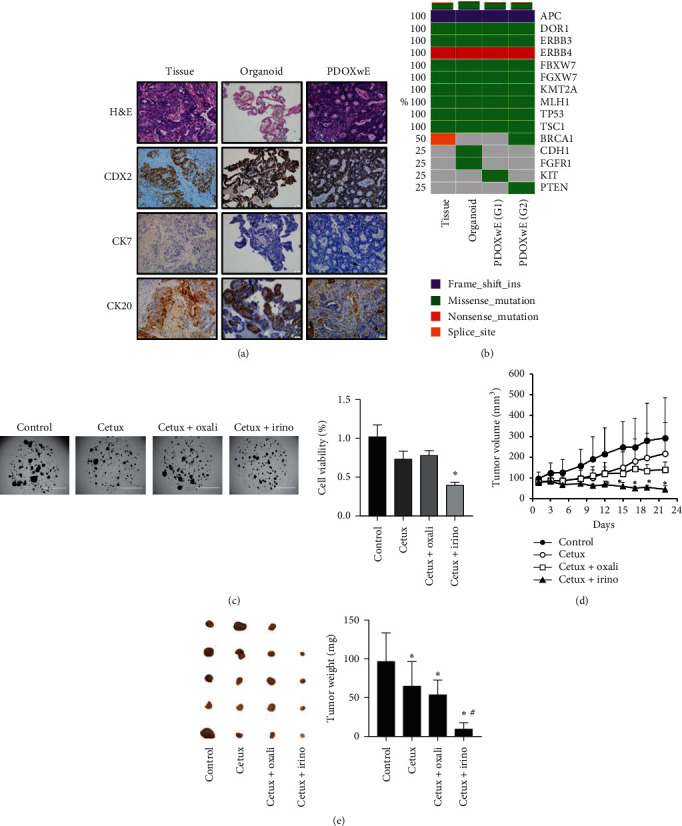
Combination therapeutic effect of cetuximab with irinotecan or oxaliplatin in organoids and PDOXwE of liver metastatic colorectal cancer. (a) Histopathology of the LMT patient's tumor tissue, organoid, and PDOXwE. Sections of the patient's tissue, patient-derived organoid, and PDOXwE were observed using H&E staining and immunohistochemistry (CDX2, CK7, and CK20). (b) Gene expression of the LMT patient's tumor tissue, organoid, and PDOXwE (each generation). The expression of fifteen major genes was analyzed by DNA sequencing. (c) Cytotoxicity of anticancer drugs in the organoid model. Cetuximab (oetux), oxaliplatin (oxali), and irinotecan (irino) (2.5 mg/mL, respectively) were used to treat the organoids for seven days. Organoids were photographed, and then, organoid cell viability was measured by the CellTiter 96 Aqueous One solution Cell Assay. ^*∗*^*p* < 0.05 (one-way ANOVA test). (d) Efficacy test of anticancer drugs in PDOXwE. Cetux (10 mg/kg, two times/week), oxali (5 mg/kg, three times/week), and irino (20 mg/kg, five times/week) were intraperitoneally injected. Tumor volumes were measured three times per week. Data are expressed as the mean ± standard deviation (*n* = 5/group). ^*∗*^*p* < 0.05 (one-way ANOVA test). (e) Comparison of tumor size and tumor weight. After measuring the final tumor volume, tumors were isolated, photographed, and weighed on a scale. ^*∗*^*p* < 0.05 (vs. control); ^#^*p* < 0.05 (vs. cetux) (one-way ANOVA test).

As shown in Figures 3(a) and 3(b), the histopathology and DNA sequence analyses demonstrate that PDO and PDOXwE also coincided with the LMT patient's tissue. The expression of several genes was different among the tissue, PDO, and PDOXwE; nevertheless, the gene profile of PDOXwE (G2) for preclinical evaluation was almost similar to that of the tissue. After seven days of observation of the PDO model, the cytotoxicity of cetuximab was not significantly enhanced; by contrast, the combination of cetuximab and irinotecan significantly enhanced cytotoxicity compared to cetuximab alone (Figure 3(c)). On the other hand, the combination of cetuximab and oxaliplatin showed no difference with the use of cetuximab alone. The tumor growth of PDOXwE was significantly suppressed only when the combination of cetuximab and irinotecan was used (Figure 3(d)). Moreover, the chemotherapeutic efficacy of PDOXwE was the same as that in the PDO model. However, tumor size and weight significantly decreased in all drug-treated groups on the final day after the 3-week treatment period (Figure 3(e)). The combination of cetuximab and irinotecan inhibited the suppression of tumor growth, tumor size, and tumor weight.

### 3.4. Monitoring of the LMT Patient Receiving the Irinotecan-Based Regimen

**Figure 4:**
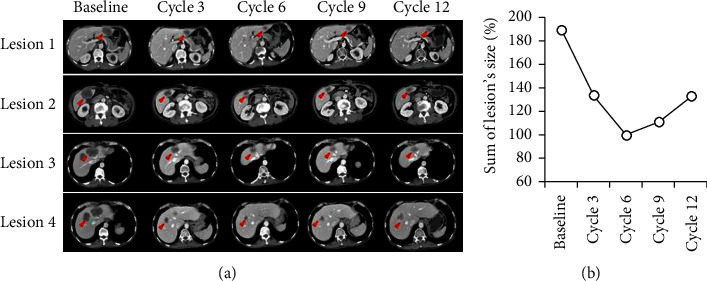
Chemotherapeutic efficacy in the LMT patient as observed by computed tomography (CT). (a) CT imaging of the LMT patient. The LMT patient received the FOLFIRI regimen, as described in Section 2. CT imaging was observed every three cycles of chemotherapy. Red arrows indicate cancer sites. (b) Sum of the lesions' sizes. The sizes of four lesions were summed, and then, percentage was calculated based on the sum of the lesion's size at cycle 6.

As shown in Figure 3, our results suggest that irinotecan is more effective than oxaliplatin in the LMT organoid and the LMT organoid-bearing xenograft models. In the LMT organoid-supplied patient, a liver metastasis of approximately 2 cm was detected at the edge of liver segment IIb. Thus, we decided to use the FOLFIRI regimen for palliative chemotherapy based on the results of preclinical tests. We monitored the chemotherapeutic efficacy every 3 cycles using CT (Figure 4(a)). Four lesions were analyzed in every detection, and the total lesion size was calculated (Figure 4(b)). The best response to chemotherapy was achieved after the 6th cycle, and the patient remained at the stable disease status until the 9th cycle. After the 12th cycle, the size of the target lesions increased by more than 20% of the size of the best response, and we determined that the disease has progressed.

## 4. Discussion

In this study, chemotherapeutic efficacy was evaluated in an *in vitro* and an *in vivo* model. Drug sensitivity of the LMT patient was extrapolated based on these results and monitored using CT.

In the preclinical test, these models were expected to predict chemosensitivity in cancer patients. PDO and PDOX models must represent some of the cancer patients' attributes (growth and gene expression); thus, PDOX exhibits different sensitivities to anticancer drugs depending on a patient's organoid (Supplementary Figure 1). Practically, time constraints are addressed to applicate the results of these preclinical assessments for cancer patients. Thus, advanced xenograft models were developed through the cotransplantation of PDO and ECFCs (Figure 1(a)). The PDOXwE model improved the period of establishment, which is a limitation in the utilization of such models for preclinical evaluation (Figure 2). Moreover, our results suggest that PDOXwE could have an edge as an *in vivo* model and, particularly, as an anticancer drug screening system for precision medicine.

PDO is emerging as a model of pathophysiology because it exhibits intratumor heterogeneity [12]. Furthermore, PDO has maintainability with long-term expansion culture [2]. Thus, PDO could be used for high-throughput screening in an *in vitro* model. PDO must be an attractive *in vitro* model for development of anticancer drugs. Nevertheless, PDO could not show tumor-stroma interaction and the integratable immune system [13]. Therefore, indirect targeted anticancer agents, such as antiangiogenic agents and inhibitors of crosstalk between cancer cells and surrounding cells, are not suitable for evaluation in PDO.

To remedy PDO's shortcomings, the evaluation of anticancer agents in an *in vivo* model was required for development of chemotherapeutic agents. Transplanted materials of xenograft models for anticancer drug screening have been developed from human cancer cell lines to PDOs [4, 14]. Xenograft models could effectively evaluate the chemotherapeutic efficacy. In general, standard protocols have been established for human-cancer-cell-derived xenograft models. Thus, this model has been used easily for a long time in the field of anticancer drug development. However, this model could not show the diverse characteristics of cancer patients [15]. The PDX model improves the obstacles of the human-cancer-cell-derived xenograft model [14]. The PDX model as an avatar model represents genetic alterations and pathohistological characteristics of cancer patients [16]. Unfortunately, this model has several limitations including long establishment period and low engraftment rate [16], which may be one of the major hurdles to apply PDX models to the effective anticancer drug screening system. As an improving model, transplantation of PDO into immunodeficient mouse has been tried. The PDOX model retains the advantages of PDX. Thus, it could predict anticancer drug susceptibility just like in patients. However, these models could only be used with some organoids. Furthermore, an optimized protocol of PDOX for stable engraftment rate and rapid establishment period is not yet found. Unsolved limitations may be due to the insufficient blood supply to the cells within the PDO after implantation. Current methodologies have been improving on previous drawbacks, with the added advantage of being able to mimic the tumor microenvironment of patients. In this study, cotransplantation with ECFCs was tried to promote quick blood vessel formation surrounding PDO. In addition to the improvement of PDO progression, a newly formed vascular network surrounding PDO can facilitate drug delivery to the PDO and also provide the screening system to estimate indirect targeted anticancer agents. Our results indicated that the time required for PDOX establishment can be reduced by ECFCs.

ECFCs are circulating endothelial progenitor cells and contribute to neovascularization in many postnatal pathophysiological conditions. For example, circulating ECFCs are recruited into the ischemic tissues, where they are incorporated into the vascular endothelial lining and differentiate into endothelial cells to form new blood vessels [17]. Furthermore, it has been reported that about 40% of vascular endothelial cells within the tumor region are derived from ECFCs originated from the bone marrow [18]. Moreover, ECFCs have adhesiveness and migratory activities toward tumor [19]. Human-originated blood vessels are made to the vasculature of xenograft models by ECFCs around PDO. Similar to our results, human-derived blood vessels could be observed in tumor tissues of a breast cancer xenograft model by coinjection of MDA-MB-231 cells and ECFCs [20]. In our study, the transplanted PDO exhibited faster tumorigenicity and tumor growth through the blood vessels newly formed by ECFCs (Figure 2). Thus, by application of ECFCs to the PDOX models, PDOXwE can be a novel strategy to establish an effective and practicable screening system for the personalized cancer medicine. Furthermore, our results showed that PDOXwE preserved patient genetic information, and some of the variations in gene expression were negligible (Figure 2). During anticancer drug screening, the drug sensitivity was observed to be coincident between PDO and PDOXwE (Figures 3(c) and 3(d)).

When the result of preclinical assessment was applied in chemotherapy, irinotecan was effective in the chemotherapy of the patient (Figure 4). These results indicated that PDO and PDOXwE models could predict chemotherapeutic efficacy in a patient.

## 5. Conclusions

In this study, PDOXwE as an advanced xenograft model was established by cotransplantation of organoids and ECFCs. The advanced xenograft model has a short establishment period and high success rate. The advanced xenograft model is an edge in preclinical modeling for precision medicine. Thus, the PDOXwE model is anticipated to be applied in precision medicine in the field of chemotherapy.

## Data Availability

The data used to support the findings of this study are included within the article and the supplementary information file.

## Supplementary Materials

Supplementary Materials Supplementary Figure 1. Sensitivity of oxaliplatin in PDOX oxaliplatin (5 mg/kg) was intraperitoneally injected in xenograft models bearing patient-derived organoids (left, 5T patient; right, 8T patient) 3 times per week for 3 weeks. Tumor size was measured 3 times per week by using a caliper. Tumor volume was calculated as follows: 0.5 × (the longest diameter) × (the shortest diameter) 2. Data were presented as mean ± standard deviation (*n* = 4/group). Significance: ^*∗*^*p* < 0.05 (Student's *t* test). .
