# Supplementary material for: Advanced Xenograft Model with Cotransplantation of Patient-Derived Organoids and Endothelial Colony-Forming Cells for Precision Medicine
Source: J Oncol. 2021 Jul 13;2021:9994535. doi: 10.1155/2021/9994535 (PMC8294990; doi:10.1155/2021/9994535)
Supplement: Supplementary Materials — Supplementary Figure 1. Sensitivity of oxaliplatin in PDOX oxaliplatin (5 mg/kg) was intraperitoneally injected in xenograft models bearing patient-derived organoids (left, 5T patient; right, 8T patient) 3 times per week for 3 weeks. Tumor size was measured 3 times per week by using a caliper. Tumor volume was calculated as follows: 0.5 × (the longest diameter) × (the shortest diameter) 2. Data were presented as mean ± standard deviation (n = 4/group). Significance: ∗p < 0.05 (Student's t test). . [file 9994535.f1.pdf]

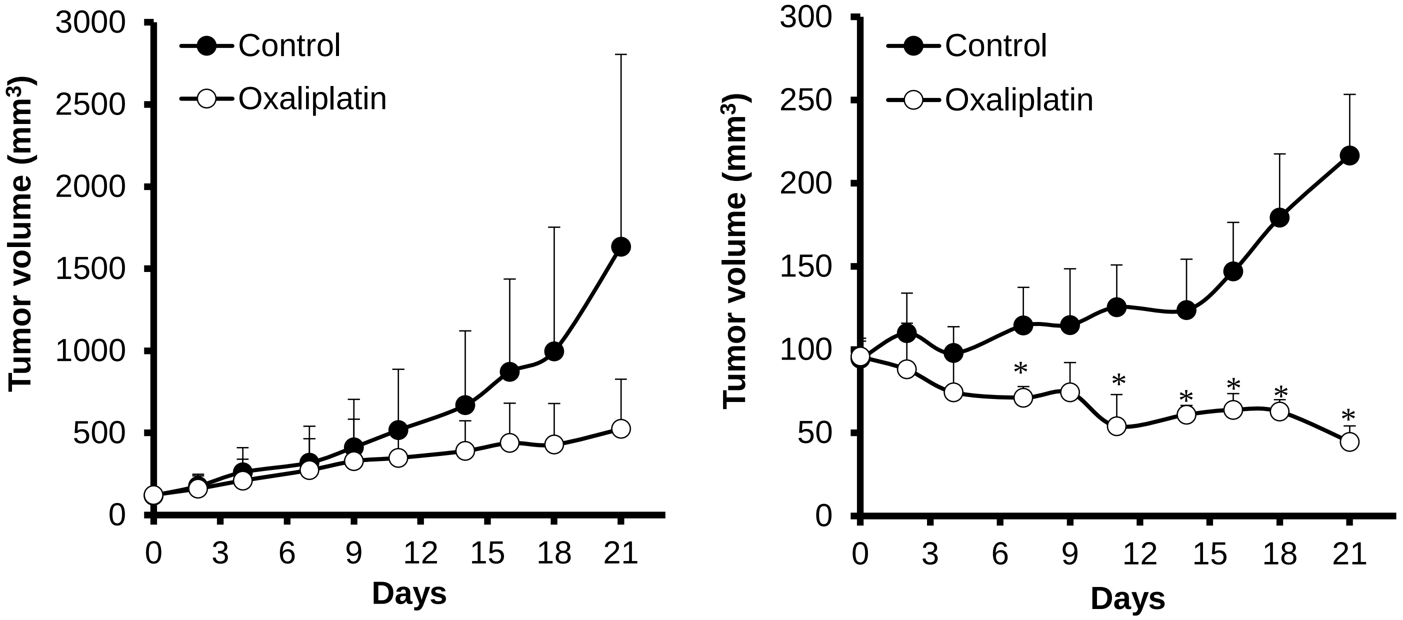

Supplementary Figure 1. Sensitivity of oxaliplatin in PDOX

Oxaliplatin (5 mg/kg) was intraperitoneally injected in xenograft models bearing patient-derived organoids (left, 5T patient; right, 8T patient) 3 times per week for 3 weeks. Tumor size was measured 3 times per week by caliper. Tumor volume was calculated following as: 0.5 × (the longest diameter) × (the shortest diameter)^2. Data presented as mean ± standard deviation (n = 4/group). Significance: *, $p < 0.05$ (Student's *t*-test).
